# Constitutive IDO expression in human cancer is sustained by an autocrine signaling loop involving IL-6, STAT3 and the AHR

**DOI:** 10.18632/oncotarget.1637

**Published:** 2014-01-20

**Authors:** Ulrike M. Litzenburger, Christiane A. Opitz, Felix Sahm, Katharina J. Rauschenbach, Saskia Trump, Marcus Winter, Martina Ott, Katharina Ochs, Christian Lutz, Xiangdong Liu, Natasa Anastasov, Irina Lehmann, Thomas Höfer, Andreas von Deimling, Wolfgang Wick, Michael Platten

**Affiliations:** ^1^ Department of Neurooncology, Neurology Clinic and National Center for Tumor Diseases University Hospital of Heidelberg, Heidelberg, Germany; ^2^ Clinical Cooperation Unit Neuroimmunology and Brain Tumor Immunology, German Cancer Research Center (DKFZ), Heidelberg, Germany; ^3^ Brain Cancer Metabolism Group, German Cancer Research Center (DKFZ), Heidelberg, Germany; ^4^ Department of Neuropathology, Institute of Pathology, University Hospital of Heidelberg, Germany; ^5^ Clinical Cooperation Unit Neuropathology, German Cancer Research Center (DKFZ), Heidelberg, Germany; ^6^ Department for Environmental Immunology, Helmholtz Center for Environmental Research, Leipzig, Germany; ^7^ Heidelberg Pharma GmbH, Ladenburg, Germany; ^8^ Incyte Corporation, Experimental Station, Wilmington, Delaware, USA; ^9^ Institute of Radiation Biology, Helmholtz Center Munich, German Research Center for Environmental Health, Germany; ^10^ Theoretical Systems Biology, German Cancer Research Center (DKFZ), Heidelberg, Germany; ^11^ Clinical Cooperation Unit Neurooncology, German Cancer Research Center (DKFZ), Heidelberg, Germany

**Keywords:** IDO, immunosuppression, autoactivation loop

## Abstract

Indoleamine-2,3-dioxygenase (IDO) inhibitors have entered clinical trials based on their ability to restore anti-tumor immunity in preclinical studies. However, the mechanisms leading to constitutive expression of IDO in human tumors are largely unknown. Here we analyzed the pathways mediating constitutive IDO expression in human cancer. IDO-positive tumor cells and tissues showed basal phosphorylation and acetylation of STAT3 as evidenced by western blotting and immunoprecipitation. Inhibition of IL-6 or STAT3 using siRNA and/or pharmacological inhibitors reduced IDO mRNA and protein expression as well as kynurenine formation. In turn, IDO enzymatic activity activated the AHR as shown by the induction of AHR target genes. IDO-mediated AHR activation induced IL-6 expression, while inhibition or knockdown of the AHR reduced IL-6 expression. IDO activity thus sustains its own expression via an autocrine AHR–IL-6–STAT3 signaling loop. Inhibition of the AHR–IL-6–STAT3 signaling loop restored T-cell proliferation in mixed leukocyte reactions performed in the presence of IDO-expressing human cancer cells. Identification of the IDO-AHR-IL-6-STAT3 signaling loop maintaining IDO expression in human cancers reveals novel therapeutic targets for the inhibition of this core pathway promoting immunosuppression of human cancers. The relevance of the IDO-AHR-IL-6-STAT3 transcriptional circuit is underscored by the finding that high expression of its members IDO, STAT3 and the AHR target gene CYP1B1 is associated with reduced relapse-free survival in lung cancer patients.

## INTRODUCTION

Evidence accumulating over the past decade indicates that activation of the tryptophan-degrading enzyme indoleamine-2,3-dioxygenase (IDO) represents a key pathway suppressing anti-tumor immunity [1, 2]. IDO is constitutively expressed by many tumors and creates an immunosuppressive microenvironment both by depletion of the essential amino acid tryptophan and by formation of immunosuppressive tryptophan metabolites such as kynurenine [3, 4]. IDO expression correlates with poor prognosis in patients with ovarian carcinoma [5], colorectal carcinoma [6] and hematological malignancies such as B-cell lymphoma [7]. In human tumors high expression of IDO is associated with reduced effector T-lymphocyte infiltration [6, 8] and increased number of regulatory T cells (Treg) [9]. Pharmacological inhibition of IDO restores anti-tumor immunity and suppresses tumor growth in preclinical models [1, 2, 10, 11] and is currently tested in clinical trials in cancer patients [12]. Preclinical models using IDO-deficient mice indicate a key role for IDO in the regulation of carcinogenesis driven by chronic inflammation [13] and in metastasis [14]. While in preclinical models the induction and expression of IDO is controlled by tumor suppressor genes such as Bin-1 [1] and oncogenes such as c-kit, respectively [8], the molecular mechanisms that drive constitutive IDO expression in human tumors are incompletely understood.

In myeloid cells, particularly in dendritic cells (DC), IDO is a key factor maintaining immune tolerance, for instance in tumor-draining lymph nodes [15]. In DC, IDO is induced through various soluble pro- and anti-inflammatory stimuli, chiefly interferon-gamma (IFN-g), and sustained by transforming growth factor-beta (TGF-b) [16]. IFN-g activates IDO1 transcription though IFN-g activating site (GAS) elements in the IDO1 promoter mediated by STAT1 phosphorylation [17]. More recently, acetylated STAT3 has been shown to transcriptionally enhance IDO expression in murine DC [18].

Signal transducer and activator of transcription 3 (STAT3) mediates a key pathway promoting tumorigenesis [19]. While constitutive STAT3 activity had initially been attributed to deregulated growth factor signaling, recent studies have identified STAT3 as an important mediator of carcinogenesis driven by chronic inflammation [19, 20]. STAT3 is constitutively active and associated with poor clinical prognosis in non-small cell lung carcinoma (NSCLC) [21], B-cell lymphoma [22] and ovarian cancer [23]. Consequently, STAT3 is an attractive target for pharmacologic intervention in cancer patients [19].

The AHR is a cytosolic transcription factor, which translocates into the nucleus upon binding of xenobiotic ligands such as benzo[a]pyrene or 2,3,7,8-tetrachlordibenzodioxin (TCDD). The AHR is involved in the formation of tumors as AHR activation enhanced clonogenic survival and motility of tumor cells [24, 25] and as transgenic mice with a constitutively active AHR spontaneously develop tumors [26].

Here, we hypothesized that the AHR and STAT3 are involved in driving IDO expression in human cancers.

## RESULTS

### IDO1 is constitutively expressed in human tumors and suppresses tumor immune cell infiltration

**Figure 1:**
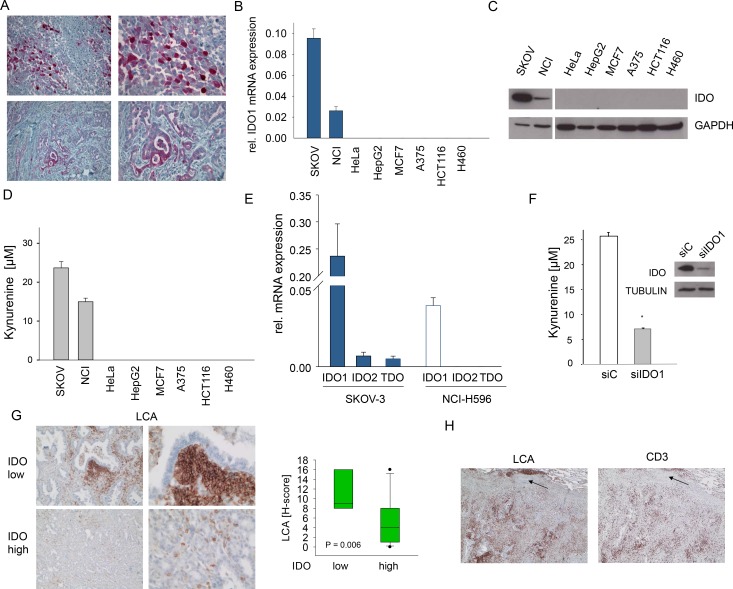
Constitutive IDO expression and activity in human cancer cells A, IDO (red) expression in ovarian carcinoma (top, representative of n=15) and NSCLC (bottom, representative of n=11). Magnification: left: 100x, right: 200x . B-D, IDO1 mRNA (B), IDO protein (C) and kynurenine release (D) of SKOV-3 ovarian carcinoma, NCI-H596 NSCLC, HeLa cervical carcinoma, HepG2 hepatocellular carcinoma, MCF7 breast cancer, A375 malignant melanoma, HCT116 colorectal carcinoma and H460 NSCLC cell lines measured by qRT-PCR, western blot and HPLC, respectively. E, Expression of the tryptophan-degrading enzymes IDO1, IDO2 and TDO in SKOV-3 and NCI-H596 cells measured by qRT-PCR. F, Kynurenine release of SKOV-3 cells after 72 h of treatment with IDO1 siRNA (siIDO1) in comparison to control (siC) measured by HPLC. IDO protein in SKOV-3 cells after 72 h of IDO1 siRNA treatment. Asterisk indicates p<0.05, error bars indicate s.e.m. G, Left: Representative immunohistochemical stainings of leukocyte common antigen (LCA) (brown) in NSCLC tissue with low or high IDO expression. Magnification: left 100x, right 200x. Right: Plot of LCA staining (H-score) in NSCLC tissues with low or high IDO expression (n=11). The H-score ranges from 0 to 300 and is calculated as the percentage of weakly stained cells plus the percentage of moderately stained cells multiplied by two plus the percentage of strongly stained cells multiplied by three. H, Immunohistochemistry illustrating the fraction of CD3+ lymphocytes among LCA+ leukocytes on consecutive sections, arrows indicate vessel for orientation. Magnification 40x.

Various cancers, including ovarian carcinoma and NSCLC express IDO (Fig 1A, Supplementary Fig 1A,B). To investigate the mechanisms underlying constitutive IDO expression, we identified from a panel of 8 human cancer cell lines two cell lines with constitutive IDO expression (Fig 1B-D). SKOV-3 ovarian carcinoma and NCI-H596 adeno-squamous lung cancer cells expressed IDO1 mRNA and IDO protein and constitutively released kynurenine into the supernatant (Fig 1B-D). In SKOV-3 and NCI-H596 IDO2 and TDO mRNA was negligible (Fig 1E). SiRNA targeting IDO1 blocked kynurenine production and IDO protein expression (Fig 1F, Supplementary Fig 1C,D). In addition, the IDO1 inhibitor 5l [30] suppressed kynurenine release (Supplementary Fig 1E). These results indicate that IDO1 is mainly responsible for the constitutive kynurenine production in SKOV-3 and NCI-H596 cells. As IDO activity has been implicated in the suppression of anti-tumor immune responses, we analyzed the effect of IDO expression on immune cell infiltration in human NSCLC. Indeed, high IDO expression was associated with a strong reduction in infiltrating leukocyte common antigen (LCA)-positive immune cells (Fig 1G, Supplementary Fig 2). Further analysis revealed that the LCA-positive immune cells included a significant amount of CD3-positive T-cells (Fig 1H). Collectively, these findings support the common notion that IDO shapes the immunological tumor microenvironment to facilitate immune evasion.

### STAT3 activity is necessary but not sufficient for constitutive IDO expression

IFN-g activates IDO expression via STAT1 [17]. We therefore analyzed STAT1 phosphorylation in SKOV-3 cells. STAT1 phosphorylation was only observed after stimulation with IFN-g but not in untreated cells (Supplementary Fig 3A). In line, knockdown of STAT1 reduced neither IDO mRNA and protein nor IDO enzymatic activity in SKOV-3 cells (Supplementary Fig 3B,C). However, treatment of SKOV-3 cells with IFN-g or polyinosinic:polycytidylic acid (pI:C), which induces interferon beta [31], further enhanced IDO expression and kynurenine release (Supplementary Fig 3D). These results suggest that STAT1 and interferons are not involved in the constitutive expression of IDO in human cancer cells but that this pathway further augments IDO activity in cancer cells with constitutive IDO activity.

**Figure 2:**
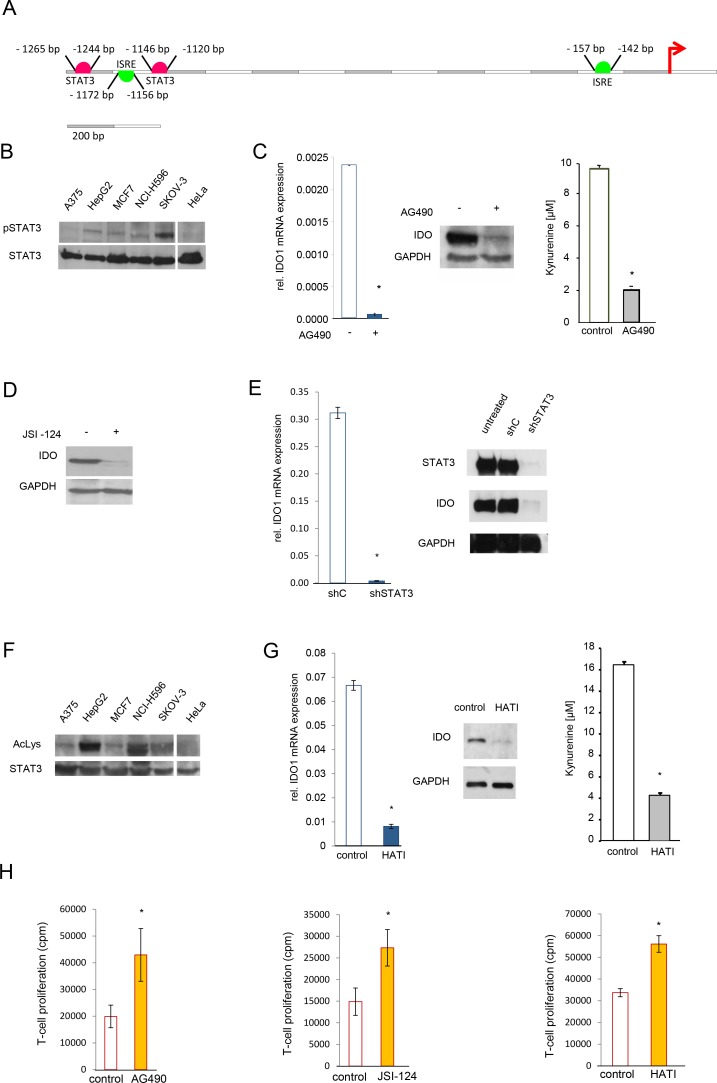
STAT3 mediates constitutive IDO1 expression *in vitro* A, Cartoon of the human IDO1 promoter showing two STAT3 binding sites (pink) and two interferon-sensitive response elements (ISRE, green) upstream of the transcription start site. B, Western blot of STAT3 phosphorylation in indicated cell lines. STAT3 served as loading control. C, IDO1 mRNA, IDO protein and kynurenine release of SKOV-3 cells after 24 h (mRNA) or 48 h (protein and kynurenine) of treatment with 100 μM AG490. D, IDO protein and kynurenine realease of SKOV-3 cells analyzed after 24 h treatment with 1 μM JSI-124. E, IDO1 mRNA and IDO protein 72 h after STAT3 shRNA in SKOV-3 cells in comparison to control. F, Immunoprecipitation of STAT3 of indicated cell lines. Acetylation of lysine residue 685 of STAT3 was analyzed using western blot. STAT3 served as loading control. G, IDO1 mRNA, IDO protein and kynurenine release of SKOV-3 cells, analyzed 24 h (mRNA) or 48 h (western blot and HPLC) after treatment with 20 μM HATI. H, Mixed leukocyte reactions (MLR) on top of 2000 SKOV-3 cells, which were pre-treated with 100 μM AG490, 1 μM JSI-124 or 20 μM HATI for 2 days before addition of the MLR in comparison to controls. Asterisk indicates p<0.05, error bars indicate s.e.m.

It has previously been reported, that STAT3 activates IDO expression in mouse DC [18, 32]. In line, analysis of the human IDO1 promoter revealed two STAT3 binding sites from -1265 bp to -1244 bp and from -1146 to -1120 as well as two ISRE from -1172 bp to -1156 and from -157 to -142 (Fig 2A). We used the ENCODE search tool to look for STAT3 chromatin immunoprecipitation DNA sequencing (ChIP-Seq) data to search for binding of STAT3 in the promoter region of IDO1. ChIP-Seq data of STAT3 of human MCF10A-ER-Src cells treated with the STAT3 activator 4-hydroxytamoxifen (1μM, 36 h; GEO sample accession, GSM935457) in comparison to the corresponding vehicle control (ethanol, 36 h; GSM935591) showed four binding sites upstream of IDO1 (Supplementary Fig 4). We chose the most pronounced binding site (Supplementary Fig 4) to further investigate possible STAT3 binding in SKOV-3 cells. Although, we determined clear site specific fold change enrichment compared to the IgG negative control, no additional increase by 4-hydroxytamoxifen treatment was detected (data not shown). This is most likely due to baseline induction of STAT3 in SKOV-3 cells, which is not further enhanced by 4-hydroxytamoxifen.

We further investigated the influence of STAT3 on IDO expression in cancer cells. STAT3 was constitutively phosphorylated in both, SKOV-3 and NCI-H596 cells, but also in IDO-negative cancer cell lines such as HepG2 hepatocellular carcinoma and MCF7 breast carcinoma cell lines (Fig 2B). To explore the contribution of STAT3 phosphorylation to constitutive IDO expression, we used tyrphostin B42 (AG490), a janus-associated kinase (JAK) inhibitor, which reduced STAT3 phosphorylation (Supplementary Fig 5A top). Inhibition of STAT3 phosphorylation reduced IDO1 mRNA, IDO protein and IDO activity (Fig 2C, Supplementary Fig 5A bottom). Similar results were obtained with the JAK inhibitor, cucurbitacin1 (JSI-124) (Fig 2D, Supplementary Fig 5B). In addition, knockdown of STAT3 decreased IDO mRNA and protein (Fig 2E, Supplementary Fig 5C). Taken together, these data suggest that STAT3 activity mediates constitutive IDO expression and activity in human cancer cells. STAT3 activity is also controlled by acetylation, which is critical for its dimerization and thus required for DNA binding [33]. Acetylation of STAT3 modulates IDO expression in murine DC [18]. STAT3 acetylation was detected in IDO-positive, as well as IDO-negative tumor cells (Fig 2F). To further investigate, if acetylation of STAT3 influences IDO expression in tumors, the CBP/p300 histone acetyltransferase complex was inhibited by a specific antagonist HATI, which resulted in reduced IDO expression and enzymatic activity (Fig 2G). While it cannot be ruled out that this inhibitor may exert additional effects by modulating histone acetylation [33], these results nevertheless indicate that constitutive acetylation as well as phosphorylation of STAT3 is involved in, but not sufficient for the constitutive expression of IDO1 in human cancer cells. In line with our data demonstrating the relevance of IDO activity for the suppression of anti-tumor immune responses and the relevance of STAT3 activation for constitutive IDO activity in human cancer, interference with constitutive STAT3 signaling in IDO-positive cancer cells using either the phospho-STAT3 inhibitors AG490 and JSI-124 or the histone acetyltransferase inhibitor HATI resulted in enhanced proliferation of allogeneic T-cells in SKOV-3/MLR cocultures (Fig 2H).

### STAT3 is phosphorylated and acetylated in IDO-expressing human tumor tissue

**Figure 3:**
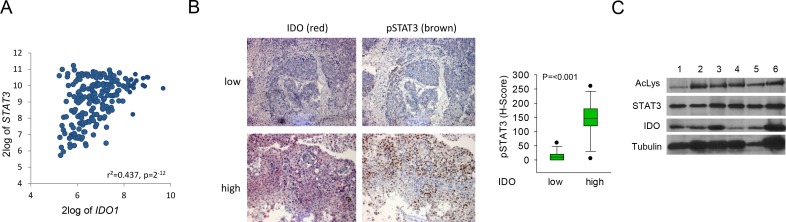
STAT3 mediates IDO1 expression *in vivo* A, Correlation of IDO1 and STAT3 expression in B-cell lymphoma [27]. B, Left: Representative immunohistochemical stainings of p-STAT3 (brown) in NSCLC metastasis tissue with low or high IDO expression. Magnification: 100x. Right: Plot of pSTAT3 staining in NSCLC metastasis with low or high IDO expression (n=27). C, Left: Immunoprecipitation of STAT3 of 6 different IDO-positive NSCLC tissue lysates. Acetylation of lysine residue 685 of STAT3 was analyzed by western blot. STAT3 served as loading control. IDO protein expression was confirmed by western blot.

Next we examined the relevance of STAT3 signaling for IDO1 expression in human tumor tissue. B-cell lymphoma is a disease with constitutively active JAK/STAT signaling [22]. Constitutive IDO activity in B-cell lymphoma is a prognostic factor of poor outcome [7, 34]. Expression analysis of 215 human mature aggressive B-cell lymphomas [27] revealed a positive correlation of IDO1 with STAT3 (Fig 3A). In addition, NSCLC tissues with high IDO expression showed enhanced STAT3 phosphorylation in comparison to tissues with low IDO expression (Fig 3B, Supplementary Fig 9). The relevance of STAT3 acetylation for IDO expression was analyzed in human fresh frozen NSCLC tissue. IDO was expressed and STAT3 was acetylated in all tumor samples examined (Fig 3C). In summary these results suggest that STAT3 activation may drive IDO expression in the analyzed tumors.

### IL-6 modulates IDO expression in human cancer via STAT3

**Figure 4:**
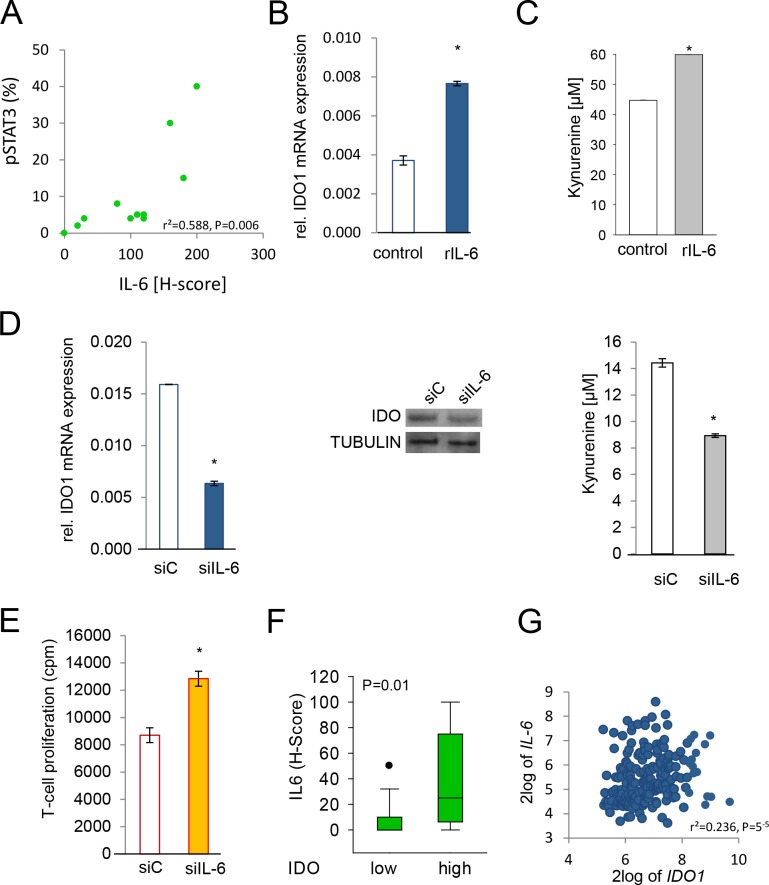
IL-6 is involved in the regulation of IDO A, Correlation of pSTAT3 and IL-6 in NSCLC tissue (n=11). B, IDO1 mRNA in SKOV-3 cells in response to 0.1 ng/ml recombinant human IL-6, analyzed after 48 h by qRT-PCR. C, Kynurenine release of NCI-H596 cells 48 h after addition of 0.1 ng/ml recombinant human IL-6 measured by HPLC. D, IDO1 mRNA in SKOV-3 48 h after knockdown of IL-6 by siRNA (siIL-6) or control (siC). IDO protein of NCI-H596 cells treated for 72 h with siC or siIL-6, analyzed by western blot. Kynurenine release of SKOV-3 cells 96 h after knockdown of IL-6 in comparison to control, measured by HPLC. E, Mixed leukocyte reactions (MLR) on top of 2000 SKOV-3 cells pre-treated with siRNA targeting IL-6 or siC for 2 days before addition of the MLR. F, Plot of IL-6 expression (H-score) in NSCLC metastasis tissue with low or high IDO expression (n=27). G, Correlation between IDO1 and IL-6 in B-cell lymphoma [27]. Asterisk indicates p<0.05, error bars indicate s.e.m.

IL-6-mediated activation of STAT3 is a key pathway promoting tumorigenesis [19]. In human NSCLC STAT3 phosphorylation correlated with IL-6 protein expression (Fig 4A). IDO-positive cells with constitutively phosphorylated STAT3 expressed IL-6 mRNA and secreted IL-6 (Supplementary Fig 6A,B). Exogenous IL-6 slightly increased IDO1 mRNA expression and kynurenine release in SKOV-3 and NCI-H596 cells (Fig 4B,C). However, IL-6 treatment did not increase STAT3 binding in the region upstream of IDO1, which might again be due to strong baseline activation of STAT3 in SKOV-3 cells. SiRNA targeting IL-6 inhibited IDO1 mRNA, IDO protein and IDO enzymatic activity in SKOV-3 and NCI-H596 cells (Fig 4D). Finally, inhibition of IL-6 production in SKOV-3 cells reduced the ability of the tumor cells to suppress mixed leukocyte reactions (Fig 4E). Immunohistochemical analyses revealed that human NSCLC metastases with high IDO expression showed enhanced IL-6 expression in comparison to tissues with low IDO expression (Fig 4F). Expression analysis in human B-cell lymphomas showed a positive correlation between IL-6 and IDO1 [27] (Fig 4G). In addition, IDO1 correlated with the expression of the members of the IL-6 signaling complex IL-6R, IL-6ST (gp300), and JAK2 [27] (Supplementary Fig 6C). Collectively, these data suggest that constitutive IL-6 release drives IDO expression in human cancers via STAT3.

As IL-6 has been demonstrated to effectively upregulate IDO in the absence of suppressor of cytokine signaling (SOCS) 3 [32], we next investigated whether IDO-expressing cells showed lower expression of SOCS3 than cells lacking IDO. However, no such inverse correlation was detected in a panel of 10 cancer cell lines and 5 untransformed cells (Supplementary Fig 7A,B). The lack of an inverse correlation between SOCS3 and IDO1 was confirmed using expression data of 215 human mature aggressive B-cell lymphomas [27] and 204 lung adenocarcinomas [28] (Supplementary Fig 7C,D). In summary, low levels of SOCS3 do not appear to be involved in the induction of IDO by IL-6 in the human cancer cells and tissues analyzed.

### IL-6 is regulated by the kynurenine receptor AHR

**Figure 5:**
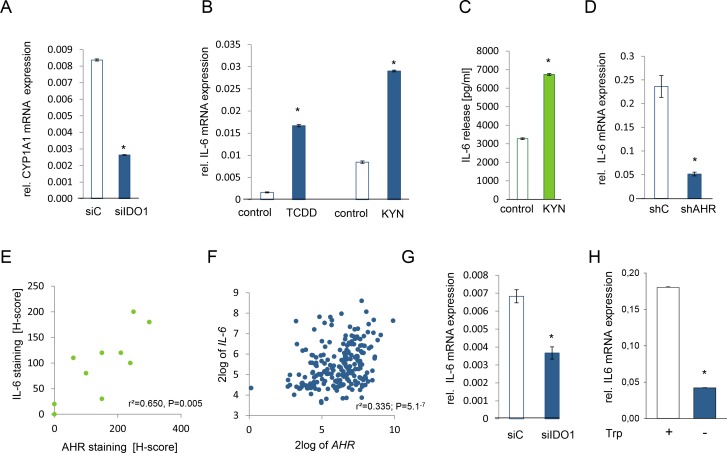
IL-6 is controlled by the kynurenine receptor AHR A, Expression of CYP1A1 in NCI-H596 cells after 24 h of IDO1 knockdown (siIDO1) in comparison to control (siC). B, IL-6 mRNA expression of tryptophan-starved NCI-H596 cells in response to 1 nM TCDD or 30 μM kynurenine (KYN), measured after 24 h. C, IL-6 secretion of tryptophan-starved NCI-H596 cells after 72 h treatment with 30 μM kynurenine (KYN), measured by ELISA. D, IL-6 mRNA expression in SKOV-3 cells with a stable AHR knockdown (shAHR) in comparison to control (shC). E, Correlation of AHR and IL-6 protein in NSCLC tissue (n=10). F, Correlation of IL-6 and AHR expression in B-cell lymphoma [27]. G, IL-6 transcript in NCI-H596 cells, measured 72 h after IDO1 knockdown (siIDO1) in comparison to control (siC). H, Expression of IL-6 in SKOV-3 cells after 120 h of tryptophan depletion in comparison to control. Asterisk indicates p<0.05, error bars indicate s.e.m.

IL-6 is transcriptionally induced via the AHR [25, 35]. Others and we have demonstrated that kynurenine activates the AHR signaling pathway in mouse immune cells [36, 37] and human brain tumor cells [25]. We thus tested the hypothesis that constitutive AHR signaling may drive IL-6 production also in other human cancer cells. Both, NCI-H596 and SKOV-3 cells expressed AHR mRNA (Supplementary Fig 8A). Kynurenine increased the expression of the AHR target genes cytochrome P450 family member 1A1 (CYP1A1), TCDD-inducible poly(ADP-ribose) polymerase (TIPARP), plasminogen activator inhibitor-2 (PAI-2), interleukin-1β (IL1B) and CYP1B1, but not of nicotinamide phophoribosyltransferase (NAMPT), which is not an AHR target and was used as negative control (Supplementary Fig 8B-G), indicating that kynurenine activates the AHR also in human ovarian carcinoma and lung cancer cells. Endogenous IDO activity was sufficient to activate the AHR in these cells, as knockdown of IDO inhibited the expression of the AHR target gene CYP1A1 (Fig 5A). As hypothesized, kynurenine enhanced IL-6 mRNA and protein to a similar degree as the classical AHR ligand TCDD (Fig 5B,C, Supplementary Fig 8H). The AHR was indeed involved in mediating IL-6 expression, as knockdown of the AHR by shRNA or addition of the AHR antagonist 3,4-DMF suppressed IL-6 transcript and protein, respectively (Fig 5D, Supplementary Fig 8I).

Next we investigated, whether IL-6 expression is mediated via the AHR in human tumors. Indeed, histological analyses revealed that IL-6 correlated with AHR expression in human NSCLC (Fig 5E). Kynurenine-mediated activation of the AHR increased AHR expression in SKOV-3 cells (Supplementary Fig 8J), indicating that AHR activation increases AHR expression. In human B-cell lymphoma IL-6 expression correlated with AHR expression [27] (Fig 5F). In addition, AHR expression correlated with the expression of the members of the IL-6 signaling complex with STAT3 binding sites in their promoters, further confirming that AHR activation is associated with IL-6 and STAT3 signaling [27] (Supplementary Fig 8K). Finally, inhibition of IDO-mediated kynurenine formation either by knockdown of IDO1 or by cultivating the cells in tryptophan-free media decreased IL-6 transcript in SKOV-3 cells (Fig 5G,H), demonstrating that endogenous IDO activity is sufficient to activate IL-6 transcription via the AHR.

### IDO sustains its own expression via an autocrine AHR–IL-6–STAT3 loop

**Figure 6:**
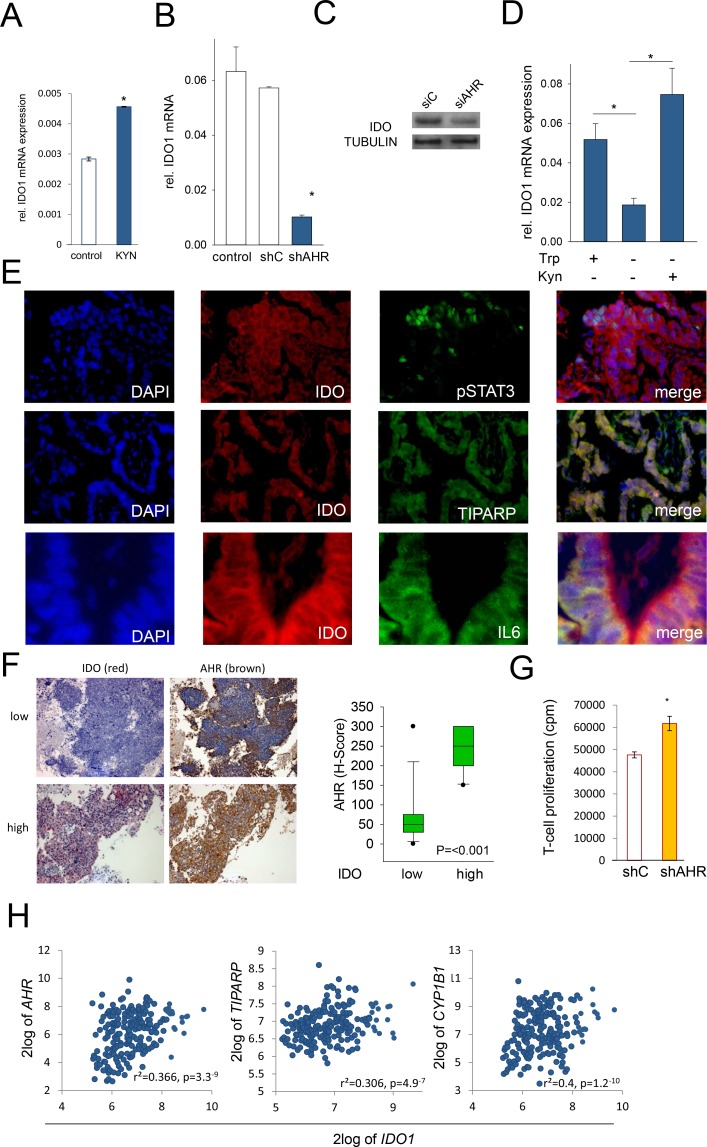
IDO sustains its expression via an autocrine AHR-IL-6-STAT3 loop A, IDO1 mRNA in NCI-H596 cells after addition of 30 μM kynurenine (KYN) to tryptophan-starved cells, analyzed after 24 h by qRT-PCR. B, IDO1 transcript in SKOV-3 cells with a stable AHR knockdown (shAHR) in comparison to controls (shC and untransfected cells). C, IDO protein 72 h after siAHR in NCI-H596 cells. D, IDO1 mRNA in SKOV-3 cells cultured in the presence of tryptophan (Trp), in the absence of Trp and kynurenine (Kyn) and in the presence of Kyn for 120 h measured by qRT-PCR. E, Immunofluorescence of IDO (red), TIPARP (green), pSTAT3 (green), IL-6 (green) and DAPI (blue) in NSCLC, magnification: pSTAT3, TIPARP 200x, IL-6 400x. F, Left: Representative immunohistochemistry of IDO (red) and AHR (brown) in NSCLC metastasis tissue with low or high IDO expression. Magnification: 100x. Right: Plot of AHR expression (H-score) in NSCLC tissue with low or high IDO expression (n=27) G, Mixed leukocyte reactions (MLR) on top of 2000 SKOV-3 cells with a stable knockdown of AHR (shAHR) or control (shC). H, Correlation between IDO1 and AHR, TIPARP or CYP1B1 expression in B-cell lymphoma [27]. Asterisk indicates p<0.05, error bars indicate s.e.m

Our data indicate that kynurenine, produced by IDO in tumor cells, activates the AHR, thereby inducing IL-6. IL-6 in turn drives IDO expression via STAT3 activation. We thus hypothesized that IDO may activate its own expression in human cancer cells via an autocrine AHR–IL-6-STAT3 signaling loop. Indeed, addition of kynurenine to NCI-H596 cells led to an increase in IDO1 mRNA expression (Fig 6A), while knockdown of the AHR reduced IDO expression in SKOV-3 cells and NCI-H596 cells (Fig 6B,C). In addition, cultivation of SKOV-3 cells in tryptophan-free media to abrogate kynurenine formation resulted in decreased IDO1 mRNA expression, which was restored by addition of exogenous kynurenine (Fig 6D). Immunofluorescence analysis revealed cellular co-expression of IDO and pSTAT3, the AHR target TIPARP and IL-6 in human NSCLC tissue (Fig 6E). Immunohistochemical analyses revealed that human NSCLC with high IDO expression showed enhanced AHR expression compared to tissue with low IDO expression (Fig 6F, Supplementary Fig 9). Furthermore, expression analysis of human B-cell lymphomas revealed a correlation of AHR and AHR target gene expression with IDO1 [27] (Fig 6H). In line, knockdown of AHR in SKOV-3 cells increased proliferation of allogeneic T-cells in tumor cell/MLR cocultures (Fig 6G). In summary, these data indicate that constitutive IDO expression in human cancer cells is sustained by its own enzymatic product - kynurenine - via an AHR–IL-6–STAT3 autoactivation loop.

### The IDO–AHR–IL-6–STAT3 loop is associated with poor prognosis in lung cancer

**Figure 7:**
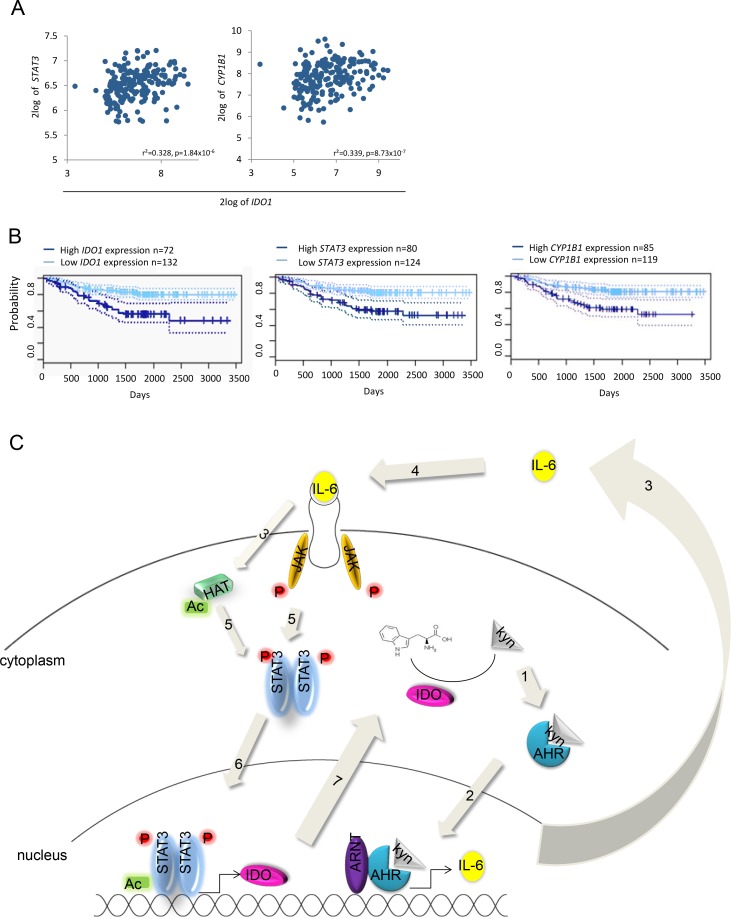
Influence of the AHR-IL-6-STAT3-IDO signaling loop on survival A, Correlation of STAT3 and CYP1B1 with IDO1 in 204 patients with stage I-II lung adenocarcinomas [28]. B, Survival probability of patients with lung adenocarcinomas with high or low IDO1, STAT3 or CYP1B1 expression. C, The IDO-AHR-IL-6-STAT3 signaling loop: In the cytoplasm, IDO metabolizes tryptophan to kynurenine (kyn). 1: Kyn activates the AHR. The AHR translocates into the nucleus (2), where it dimerizes with ARNT and binds to dioxin responsive elements (DRE) in the promoter of target genes like IL-6. 3: IL-6 is released from the cells and binds to its receptor (4). 5: Upon activation of the IL-6 receptor, Janus Kinases (JAK) phosphorylate (P) STAT3. Simultaneously, histone acetyltransferases (HAT) acetylate STAT3. Phosphorylated and acetylated STAT3 dimers translocate into the nucleus (6), where they bind to the promoter of target genes like IDO1 (7).

We next assessed the effects of the IDO-AHR-STAT3 loop in lung cancer patients. In a patient cohort of 204 lung adenocarcinoma patients [28], STAT3 and CYP1B1 expression correlated with IDO1 expression (Fig 7A). Most importantly, high IDO1, STAT3 and CYP1B1 expression was associated with reduced relapse-free survival in lung carcinoma patients (Fig 7B). These data illustrate the clinical relevance of the identified IDO–AHR–IL-6–STAT3 autoactivation loop (Fig 7C) and further suggest this transcriptional loop as a cancer drug target.

## DISCUSSION

Constitutive IDO activity in tumors is a key mechanism mediating immune evasion and thus represents an attractive therapeutic target to restore anti-tumor immunity [38]. Based on preclinical animal models demonstrating efficacy of pharmacologic IDO inhibition using 1-methyl-tryptophan (1-MT) [1, 2, 10], clinical trials with IDO inhibitors in patients with solid cancer are ongoing. Whether IDO expressed in tumors or dendritic cells is the key target for the clinically used D-stereoisomer of 1-MT is unclear, but data from preclinical studies using mouse and human tumors indicate that host IDO expression is required for the therapeutic effect of D-1-MT [10] and that D-1-MT may even increase tumoral IDO1 expression due to off-target effects [39]. While the factors and signaling events leading to expression and activity of IDO in myeloid cells have been thoroughly addressed in the past years, the events that drive constitutive IDO expression and activity in tumor cells themselves are still unclear. Current evidence suggests that IDO is a general trait of cancer signified by common genetic alterations driving tumorigenesis [40]. For instance, loss of tumor suppressor genes such as Bin-1 in tumors drives IDO induction via STAT1 and nuclear factor kB [1], two signaling molecules that have been identified to be key for the inducible IDO expression in myeloid and mesenchymal cells via IFN-g and toll-like receptor (TLR) ligands [31]. The oncogene c-Kit drives IDO expression in gastrointestinal stromal tumors (GIST) via the mammalian target of rapamycin (mTOR) and the transcription factor ETV4 [8]. In addition to mTOR signaling, inhibition of oncogenic KIT signaling suppressed STAT3 phosphorylation in GIST [8]. Based on our data demonstrating that STAT3 activity is functionally associated with IDO expression in human tumors STAT3 may be involved in regulating IDO expression stimulated by oncogenic KIT signaling. A firm connection between STAT3 activity and IDO expression has been established in mouse DC [18, 32], indicating similar patterns of transcriptional regulation of IDO in tumor cells and myeloid cells. Indeed, STAT1, a major transcriptional activator of IDO in myeloid cells, also potently induces IDO transcription in tumor cells when activated constitutively [1] or after stimulation with IFN-g (Supplementary Fig 3A,B). It is conceivable that the recently discovered pathways for sustained IDO expression in plasmacytoid DC (pDC) for instance by TGF-b [16], may also be active in cancer cells. Similar to what we observed in constitutively IDO-expressing tumor cells (Fig 7C), IDO maintains its own expression in pDC [16].

The tryptophan metabolites kynurenic acid and kynurenine were recently identified as AHR ligands in human tumor cells [25, 41]. While exogenous kynurenine is capable of subverting the proinflammatory phenotype of naïve T-cells and immature DC in mice via the AHR [36, 37], we have recently demonstrated that kynurenine is produced by human gliomas in concentrations sufficient to enhance clonogenic survival and motility of tumor cells in an AHR-dependent fashion [25]. While kynurenine is produced by the tryptophan catabolizing enzyme TDO in several types of cancer including glioma, hepatocellular carcinoma and melanoma to promote immune evasion [25, 42], we demonstrate here that IDO1 is equally capable of employing the AHR pathway in NSCLC and ovarian carcinoma cells.

We have identified a signaling pathway sustaining IDO expression via an autocrine positive feedback loop involving IL-6 through activation of the AHR by IDO-derived kynurenine. Recently, IDO was demonstrated to drive IL-6 production in mice with lung cancer and breast cancer metastasis [14], while IL-6 in turn was reported to induce IDO expression via JAK/STAT signaling in rat hippocampus [43]. Interestingly, AHR signaling has been shown to be required for the expression of IDO in mouse DC [37, 44, 45]. We may have identified the link between these observations as our results reveal the existence of an IDO-AHR-IL-6-STAT3 autoactivation loop in human cancer cells, which may underlie the induction of IL-6 by IDO in mouse tumors (via the AHR), the induction of IDO by IL-6 in rat hippocampus (via phosphorylated and acetylated STAT3) and the requirement of the AHR for IDO expression in mouse DC.

The IDO-AHR-IL6-STAT3 transcriptional loop represents a rather complex mode of positive autoregulation. Regardless of the number of components involved, positive feedback can create a bistable system [46]. The system can be in a stable off-state, in which all components of the feedback loop have only basal activity below the threshold for self-amplification, or in a stable on-state, in which the high activity of all components is self-sustained by positive feedback and limited only through constitutive degradation and inactivation reactions. The transition from the off-state to the on-state would then be a defining step in oncogenic transformation [47]. In principle, this transition could be triggered at any level of the positive feedback loop. Local inflammation presents a likely candidate as IFN-g produced by cancer-infiltrating lymphocytes may activate IDO and initiate the positive feedback loop, thus resulting in the spread of IDO expression throughout the tumor. Given the importance of IL-6-mediated STAT3 activation in carcinogenesis driven by chronic inflammation [47], the activation of the IDO-AHR-IL-6-STAT3 autoactivation loop in response to inflammatory stimuli may represent an as yet unknown connection between inflammation and carcinogenesis.

While we show that constitutively active STAT3, which is controlled by autocrine IL-6 through phosphorylation and acetylation, is required for sustained IDO expression it does not seem to be sufficient as there is constitutive STAT3 phosphorylation and acetylation in IDO-negative tumors cells and tumors. It is likely that a second signal is required to induce IDO expression, although the nature of this signal remains elusive. As proposed for the tumor suppressor gene Bin-1 [1], NF-KB may constitute the second signal required for constitutive IDO expression in cancer.

Our findings imply that therapeutic intervention at the IDO-AHR-IL-6-STAT3 loop may revert immune suppression mediated by IDO. In fact, several drugs targeting this pathway are in development or used in the clinic for the treatment of cancer including neutralizing anti-IL-6 antibodies [48] and small molecule or peptide inhibitors of STAT3 and/or JAK2 [49]. Small molecule screens to identify AHR antagonists are underway. Therapeutic targeting of STAT3 has been shown to revert cancer-associated immune suppression [20]. It is tempting to speculate that inhibition of IDO contributes to this immune-stimulatory effect. The discovery of the IDO-AHR-IL-6-STAT3 regulatory loop active in some cancers may guide future therapeutic approaches and read-out strategies for their immunotherapeutic efficacy.

## MATERIALS AND METHODS

### Cells and reagents

The cell lines used are of human origin and purchased from the American Type Culture Collection (ATCC, Rockville, MD, USA). Further details including culture conditions and reagents are provided in the Supplementary Materials and Methods.

### High performance liquid chromatography (HPLC)

HPLC analyses were performed using a Beckman HPLC with photodiode array (PDA) detection and Lichrosorb RP-18 column (250 mm x 4 mm ID, 5 μm, Merck, Darmstadt, Germany). Further details are provided in the Supplementary Materials and Methods.

### Quantitative (q)RT-PCR, siRNA and shRNA experiments

Relative quantification of gene expression was determined by comparison of threshold values. For siRNA experiments SMART-pool siRNA by Dharmacon RNA Technologies (Lafayette, CO, USA) was used. ON-TARGETplus siCONTROL Non-targeting Pool (D-001810-10-05, Dharmacon) and a transfection without siRNA were used as negative controls. AHR and STAT3 in SKOV-3 cells were knocked down using vectors coding for the respective shRNA, vectors coding for non-targeting shRNA were used as controls. Further details, including the primer sequences, siRNA and shRNA sequences are provided in the Supplementary Materials and Methods.

### Immunohistochemistry

LCA, pSTAT3, IL-6, and AHR staining was quantified using the H-score, which ranges from 0 to 300 and is calculated as the percentage of weakly stained cells plus the percentage of moderately stained cells multiplied by two plus the percentage of strongly stained cells multiplied by three. The score was assessed in a 200x magnification field of the area presenting with highest IDO1 expression and of an IDO1 negative area on consecutive sections of each case. Cases homogenously positive or negative were only assessed in one area. Details concerning specimens and staining procedures are provided in the Supplementary Materials and Methods.

### Cocultures of tumor cells and mixed leukocyte reactions (MLR)

MLR consisting of 2 * 105 irradiated (30 Gy) PBMC as stimulators and 2 * 105 PBMC from unrelated donors as responders were cocultured with 2000 SKOV-3 cells or NCI-H596 cells. After 6 days cultures were pulsed with [3H]-methylthymidine for the last 18 h, the cells were harvested, and radionuclide uptake was measured. Detailed procedures are provided in the Supplementary Materials and Methods.

### Correlation analysis

For correlation analyses of gene expression in mature aggressive B-cell lymphomas (GEO accession number GSE4475) [27] normalised Affymetrix gene expression data were downloaded from the R2 microarray analysis and visualisation platform. Survival data from NSCLC patients [28] are based on the GEO dataset GSE31210 and were extracted using the PrognoScan database [29]. Datasets are provided in the Supplementary Materials and Methods.

## SUPPLEMENTARY FIGURES, MATERIALS AND REFERENCES


