# Supplementary material for: Constitutive IDO expression in human cancer is sustained by an autocrine signaling loop involving IL-6, STAT3 and the AHR
Source: Oncotarget. 2014 Jan 20;5(4):1038–51. doi: 10.18632/oncotarget.1637 (PMC4011581; doi:10.18632/oncotarget.1637)
Supplement: Supplementary file 1 [file oncotarget-05-1038-s001.doc]

**Supplement**

**Constitutive IDO expression in human cancer is sustained by an autocrine signaling loop involving IL-6, STAT3 and the AHR**

**Inventory of Supplement:**

**9 Supplementary figures with legends**

**Supplementary Materials and Methods**

**Supplementary References**


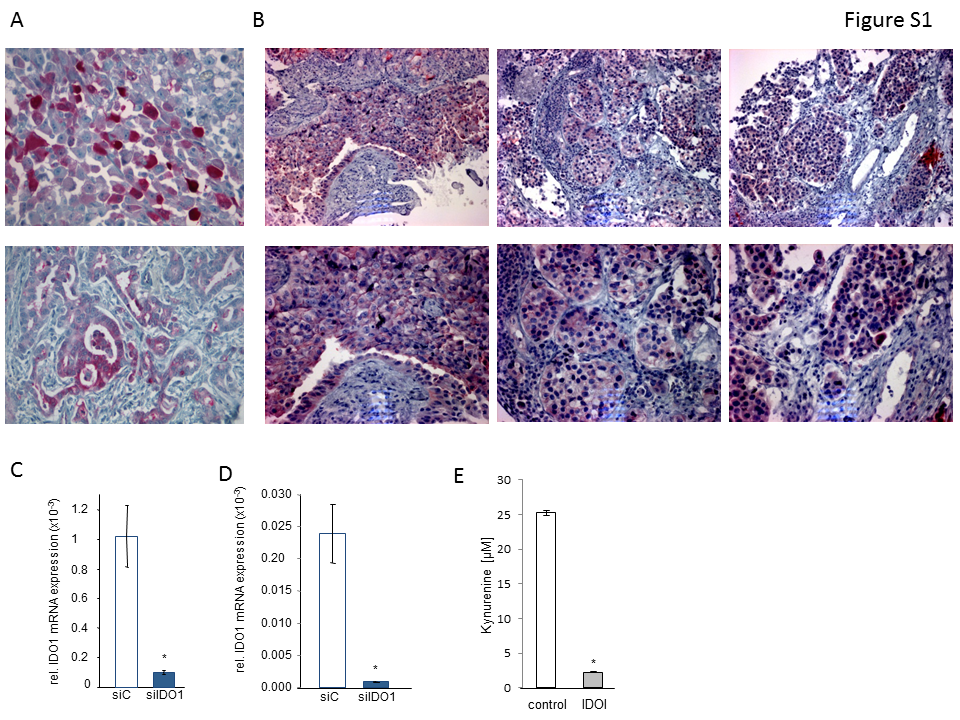


**Supplementary Figure 1**

A, Representative images of IDO (red) expression in ovarian carcinoma (top) and NSCLC bottom. Magnification: 200x. B, Representative images of IDO (red) expression NSCLC brain metastasis (representative of n=12), Magnification top: 100x Magnification bottom: 200x. C, *IDO1* mRNA expression after 24 h treatment with siRNA targeting *IDO1* (blue bar) in comparison to control (white bar) in SKOV-3 cells, measured by qRT-PCR. D, *IDO1* mRNA expression after 24 h treatment with siRNA targeting *IDO1* (blue bar) in comparison to control (white bar) in NCI-H596 cells, measured by qRT-PCR. E, Kynurenine release of NCI-H596 cells after 72 h treatment with the IDO-inhibitor 5l (IDOI) in comparison to control.

**
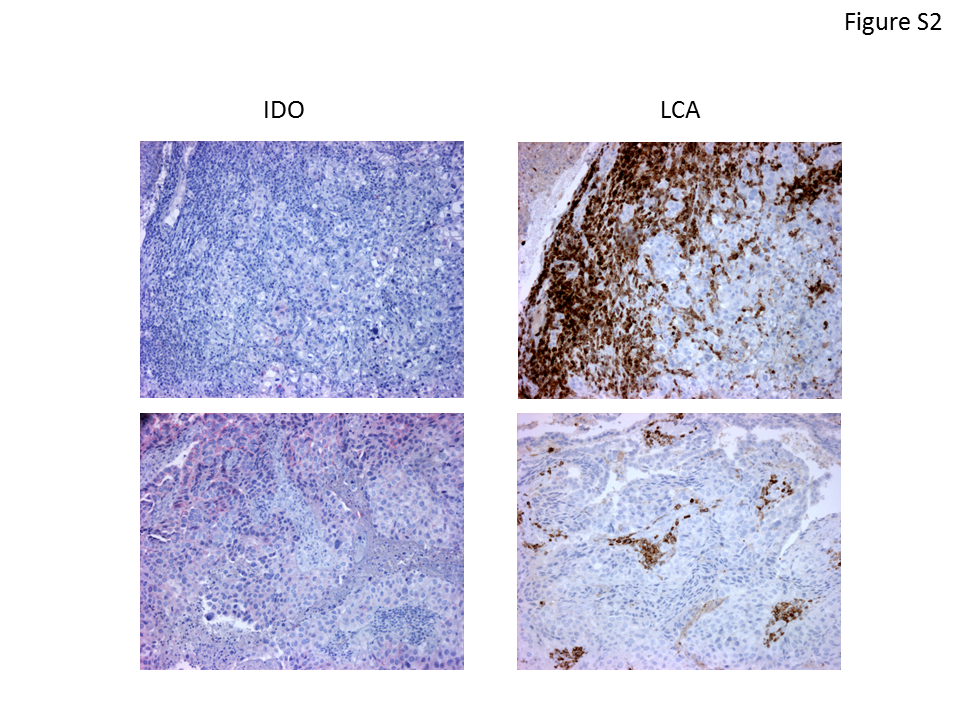
**

**Supplementary Figure 2**

Representative images of IDO (red) expression and leukocyte common antigen (LCA) staining (brown) in NSCLC metastasis tissue. Upper panel: weak IDO expression, lower panel: moderate IDO expression. Representative for n=27. Magnification: 100x.

**
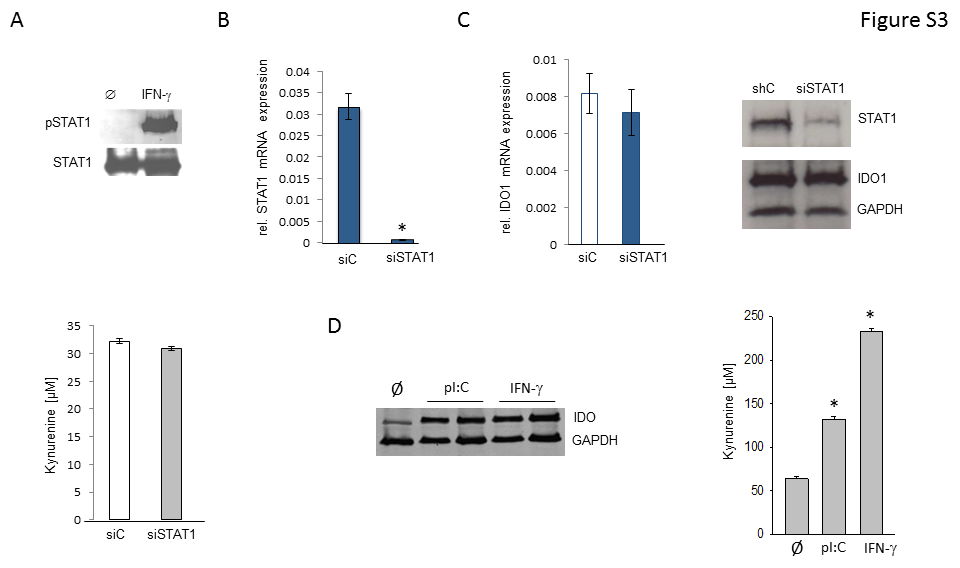
**

**Supplementary Figure 3**

A, Detection of STAT1 phosphorylation in untreated SKOV-3 cells and SKOV-3 cells stimulated with 200 U IFN-g for 24 h. Total STAT1 protein served as loading control. B, Knockdown of STAT1 by siRNA measured after 24 h by qRT-PCR. C, *IDO1* mRNA and protein expression as well as kynurenine release of SKOV-3 cells after knockdown of *STAT1* measured after 24 h (mRNA) and 48 h (protein and kynurenine). D, IDO protein and kynurenine release of SKOV-3 cells after treatment with 50 µg and 100 µg pI:C and IFN-g, respectively (24 h).

**
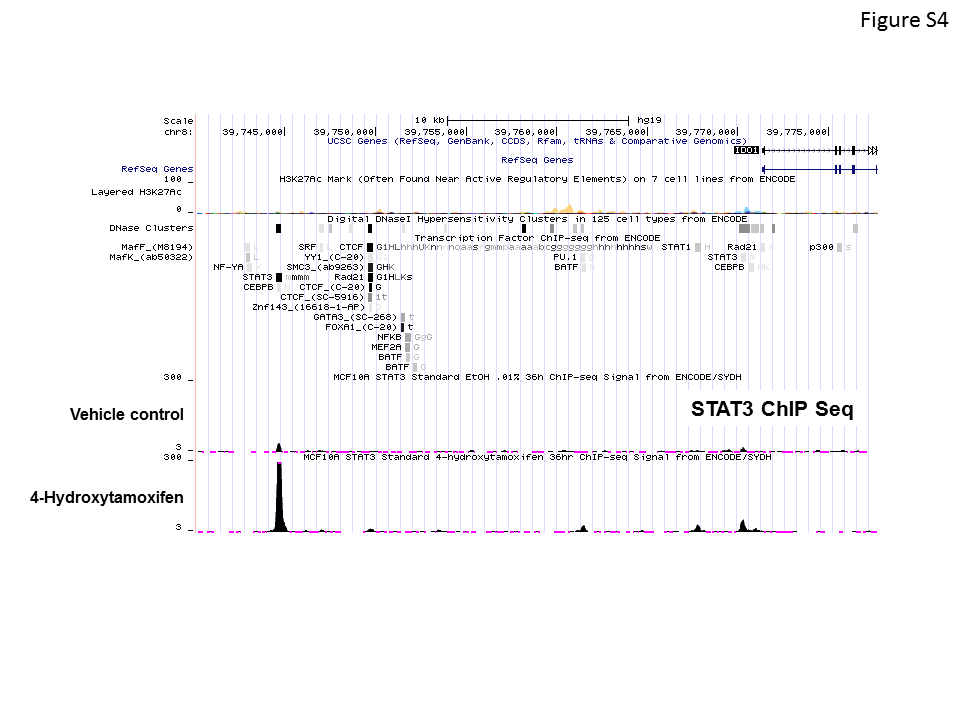
**

**Supplementary Figure 4**

ChIP-Seq data derived from the ENCODE database showing binding of STAT3 to 4 binding sites upstream of IDO1 in human MCF10A-ER-Src cells treated with the STAT3 activator 4-hydroxytamoxifen (1 µM, 36 h; GEO sample accession, GSM935457) in comparison to the corresponding vehicle control (ethanol, 36 h; GSM935591).

**
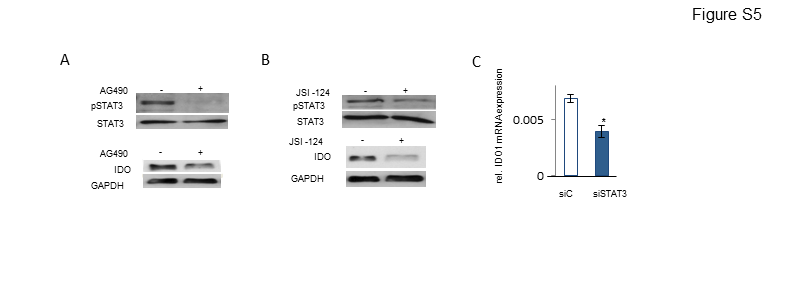
**

**Supplementary Figure 5**

A, top: Detection of STAT3 phosphorylation of NCI-H596 cells 3 h after addition of 100 µM AG490. STAT3 served as loading control. Bottom: IDO protein expression of NCI-H596 cells analyzed after 48 h treatment with 100 µM AG490 by western blot. GAPDH served as loading control. B, top: Detection of STAT3 phosphorylation of NCI-H596 cells 3 h after addition of 1 µM JSI-124. STAT3 served as loading control. Bottom: IDO protein expression of NCI-H596 cells analyzed after 48 h treatment with 1 µM JSI-124 by western blot. GAPDH served as loading control. C, *IDO1* mRNA expression in NCI-H596 cells after 48 h of specific knockdown of *STAT3* by siRNA (blue bar) relative to control (white bar).

**
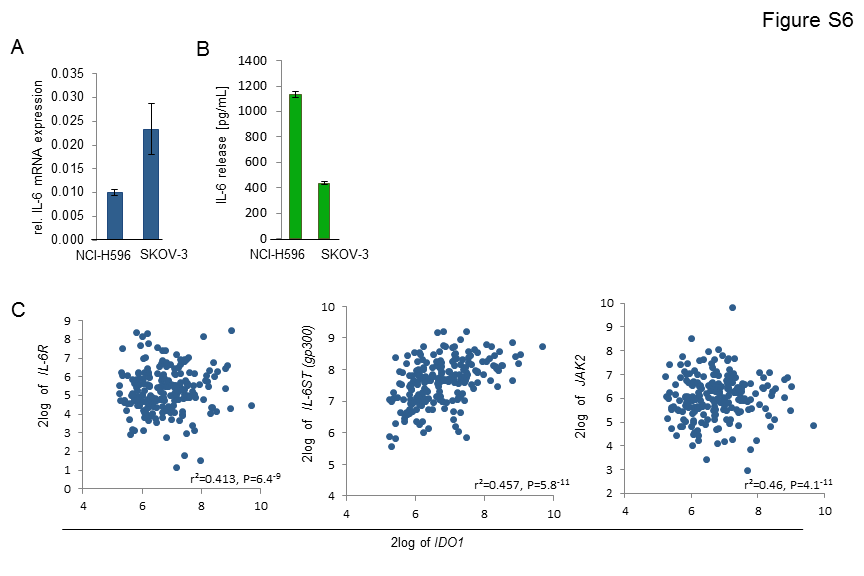
**

**Supplementary Figure 6**

A, Expression of *IL-6* mRNA in NCI-H596 and SKOV-3 cells, measured by qRT-PCR. B, Measurement of IL-6 secretion of NCI-H596 and SKOV-3 cells by ELISA. C, Correlation of the expression of *IDO1* with *IL-6R*, *IDO1* with *IL-6ST* (*gp300*) and *IDO1* with *JAK2* in B-cell lymphoma specimen (r²=0.413, p=6.4x10^-9^; r²=0.457, p=5.8x10^-11^ and r²=0.46, p=4.1x10^-11^).


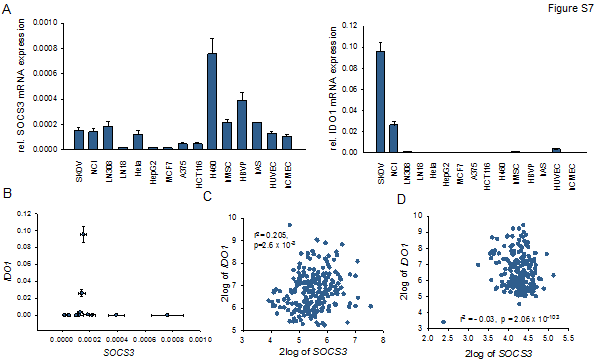


**Supplementary Figure 7**

A, *SOCS3* (left) and *IDO1* (right) mRNA expression in SKOV-3 ovarian carcinoma, NCI-H596 NSCLC, LN308 and LN18 malignant glioma, HeLa cervical carcinoma, HepG2 hepatocellular carcinoma, MCF7 breast cancer, A375 malignant melanoma, HCT116 colorectal carcinoma and H460 NSCLC cell lines as well as in non-malignant cells such as hMSC human bone marrow derived mesenchymal stem cells, HBVP human brain vascular pericytes, hAS CRL-8621 human astrocytes, HUVEC human umbilical vein endothelial cells and hCMEC, HCMEC/D3 human cerebral microvascular cells. B, Plot of IDO1 versus SOCS3 in the above mentioned cells. C, D, Plot of SOCS3 versus IDO1 using expression data of 215 human mature aggressive B-cell lymphomas (C) and 204 lung adenocarcinomas (D).

**
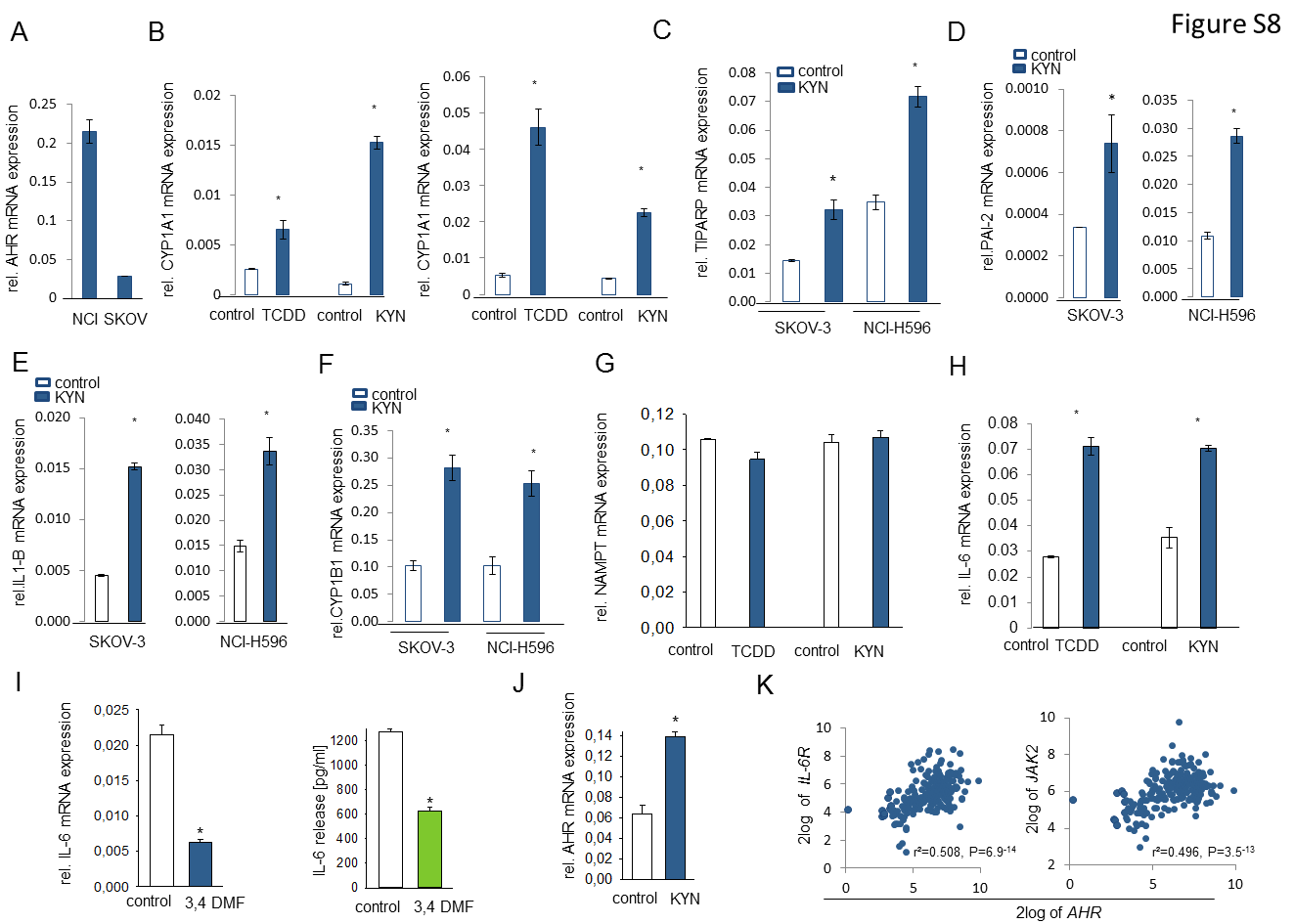
**

**Supplementary Figure 8**

A, *AHR* mRNA expression in NCI-H596 (NCI) and SKOV-3 (SKOV) cells, measured by qRT-PCR. B, Left: Expression of *CYP1A1* mRNA in SKOV-3 cells after 24 h treatment with 1 nM TCDD or 30 µM kynurenine (KYN) (blue) in comparison to control (white), measured by qRT-PCR. Right: Expression of *CYP1A1* after 24 h treatment with 1 nM TCDD or 30 µM kynurenine (KYN, blue bars) in comparison to control (white bars) in NCI-H596 cells, measured by qRT-PCR. C–F, Expression of the AHR target genes *TIPARP* (C), *PAI-2* (D), *IL1B* (E) and *CYP1B1* (F) in untreated SKOV-3 and NCI-H596 cells (white bars) or after 24 h treatment with 30 µM kynurenine (KYN, blue bars). G, Expression of NAMPT, which is not an AHR target gene and was used as negative control in SKOV-3 cells in response to 1 nM TCDD or 30 µM kynurenine (KYN) (blue) in comparison to control (white). H, *IL-6* mRNA expression in SKOV-3 cells in response to 1 nM TCDD or 30 µM kynurenine (KYN) (blue) in comparison to control (white). I, IL-6 release of NCI-H596 cells 72 h after addition of the AHR antagonist 3,4-DMF (green bar) in comparison to control (white bar). J, *AHR* mRNA expression after treatment of SKOV-3 cells with 30 µM Kyn for 24 h measured by qRT-PCR. K, Correlation of *AHR* expression with *I-L6R* expression (left) and *JAK2* expression (right) in B-cell lymphoma (r²=0.508, p=6.9x10^-14^ and r²=0.496, p=3.5x10^-13^).

**
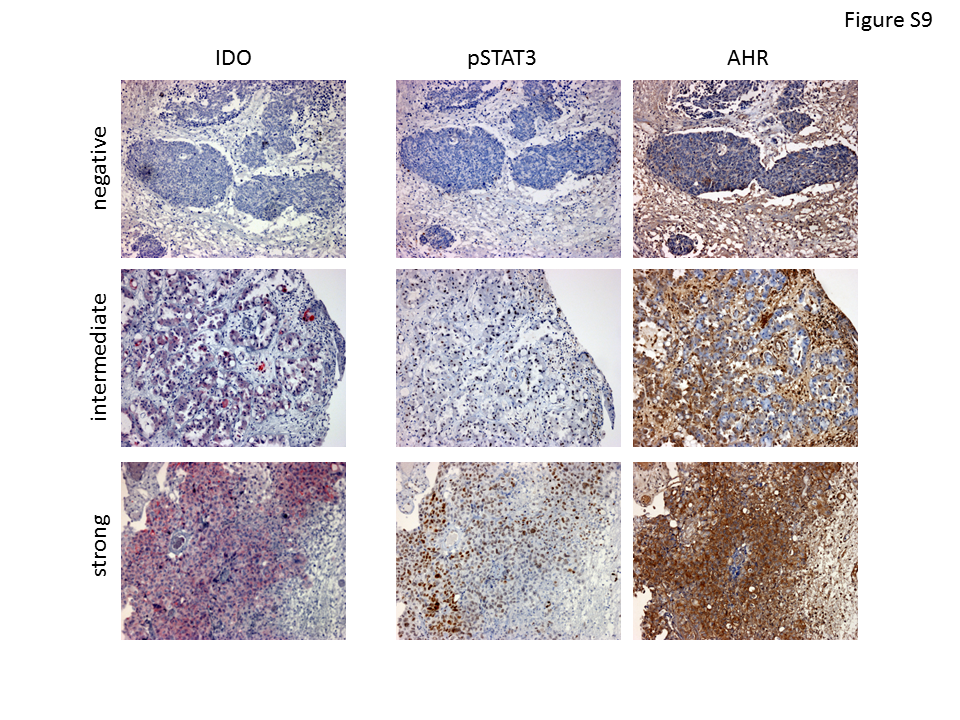
**

**Supplementary Figure 9**

Representative images of IDO protein expression (red) and corresponding pSTAT3 (brown) as well as AHR (brown) protein expression in NSCLC metastasis. Representative for n=27. Magnification: 100x.

**Supplementary Materials and Methods**

Cells and reagents

Ovarian carcinoma cells SKOV-3, NCI-H596 NSCLC cells, A375 malignant melanoma cells, MCF7 breast cancer cells, HeLa cervical carcinoma cells HepG2 hepatocellular cells, HCT116 colorectal carcinoma cells, H490 NSCLC cells, LN18 malignant glioma cells are of human origin and were purchased from the American Type Culture Collection (ATCC, Rockville, MD, USA). The LN308 glioma cell line was kindly provided by Dr. N. de Tribolet (Lausanne, Switzerland). The cancer cell lines were authenticated in November 2013. A375, MCF7, HeLa, HepG2, HCT116, H490, LN18, LN308 cells and CRL-8621 human astrocytes were cultured in Dulbecco’s modified Eagle’s medium (DMEM, PAA Laboratories, Pasching, Austria) supplemented with 10% FBS (Thermo Fisher Scientific Inc., Waltham, MA, USA) and 100 U/ml penicillin and 100 μg/ml streptomycin (PAA Laboratories). SKOV-3 were cultured in McCOY’s 5A Medium (BioConcept, Allschwil, Switzerland) supplemented with 10% FBS (Thermo Fisher Scientific Inc.) and 100 U/ml penicillin and 100 μg/ml streptomycin (PAA Laboratories). NCI-H596 cells were kept in RPMI 1640 (PAA Laboratories), supplemented with 10% FBS and 100 U/ml penicillin and 100 μg/ml streptomycin. Human MSC were obtained from bone marrow from total hip replacement surgeries following informed consent . After density gradient centrifugation, MSC isolated by plastic adherence were grown in Amniomax Basal Medium (AM) with 10% stimulatory supplement (Invitrogen Life Science, Carlsbad, USA, http://www.invitrogen.com). Passages 5-20 were used for experiments.

HBVP were purchased from ScienCell Research Laboratories (Carlsbad, USA, www.sciencellonline.com) and cultured in poly-L-lysine coated flasks in basal medium for human vascular pericytes (PM) containing 2% fetal bovine serum (FBS), 1% pericyte growth supplement and 1% penicillin/streptomycin solution (all reagents from ScienCell Research Laboratories). Passages 3-15 were used for experiments.

Immortalized human cerebral microvascular endothelial cells (HCMEC/D3) were cultured in rat tail collagen type-1 (Sigma-Aldrich, Taufkirchen, Germany, www.sigmaaldrich.com) coated dishes in Clonetics EBM-2 Endothelial Cell Basal Medium-2 containing 5% FBS, 1% penicillin/streptomycin, 1,4 μM hydrocortisone, 5 μg/ml acid asorbic, 1% chemically defined lipid concentrate, 10 mM HEPES and 1 ng/ml bFGF (all reagents from Lonza, Basel, Switzerland, www.lonza.com). Passages 25-35 were used for experiments.

Primary human umbilical vein endothelial cells (HUVEC) were purchased from PromoCell and kept in endothelial cell growth medium (ECGM) supplemented with Supplement Mix C-39215 (PromoCell), 10% FBS, 100 IU/mL penicillin, and 100 mg/mL streptomycin.

For comparison analyses all the cells were kept in same medium (DMEM).

For tryptophan (trp)-free experiments SKOV-3 cells were seeded in trp-free McCOY’s 5A Medium (BioConcept), NCI-H596 cells in trp-free RPMI 1640 (PAN biotech, Aidenbach, Germany) supplemented with dialyzed FBS (Life Technologies, Darmstadt, Germany) and 100 U/ml penicillin and 100 μg/ml streptomycin. All cells were routinely tested for contamination by multiplex cell **contamination** test . Cultures were incubated at 37°C in a 5% CO_2_ atmosphere. L-tryptophan and kynurenine were purchased from Sigma-Aldrich (Taufkirchen, Germany). Recombinant human IL-6 and human recombinant IFN-γ were purchased from Immunotools (Friesoythe, Germany). 2,3,7,8-Tetrachlordibenzodioxin (TCDD) was kindly provided by D. Schrenk (Kaiserslautern, Germany) and 3,4-dimethoxylflavone (3,4-DMF) was obtained from Alfa Aesar (Karlsruhe, Germany). The IDO inhibitor 5l was synthesized as recently described . The histone acetylase inhibitor (HATI) , a *bis*-arylidene cyclohexanone compound and the JAK inhibitors AG490 (tyrphostin B42) and JSI-124 (cucurbitacin1) were purchased from Calbiochem (Darmstadt, Germany). The experiments with HATI, AG490 and JSI-124 were performed in serum free media.

High performance liquid chromatography (HPLC)

HPLC analyses were performed using a Beckman HPLC with photodiode array (PDA) detection and Lichrosorb RP-18 column (250 mm x 4 mm ID, 5 µm, Merck, Darmstadt, Germany). Kynurenine and tryptophan concentrations were measured in the medium of 3 x 10^5^ cells in 2 ml medium containing 10% FBS 100 U/ml penicillin and 100 μg/ml streptomycin. The medium was harvested from 6 well plates after indicated time points, centrifuged and frozen until further analysis. After thawing, the samples were supplemented with trichloroacetic acid for protein precipitation, centrifuged and 100 µl of the supernatant was analyzed by HPLC. Standard curves were generated with kynurenine and tryptophan in the same medium. Since FBS contains kynurenine, low kynurenine concentrations (~1 µM) were detected in all samples and medium without cells was always measured for comparison. Each sample was measured at least in duplicate.

# Quantitative (q)RT-PCR

Total RNA was isolated with the Qiagen RNAeasy RNA isolation kit (Hilden, Germany) and cDNA was synthesized with the Applied Biosystems reverse-transcription-Kit (Foster City, CA, USA). QRT-PCR was performed in an ABI 7000 thermal cycler with SYBR Green PCR Mastermix (Applied Biosystems). All primers were separated by at least one intron on the genomic DNA to exclude amplification of genomic DNA. PCR reactions were checked by including no-RT-controls, by omission of templates and by both melting curve and gel analysis. The size of the amplicons was analyzed by loading the samples and a 100 bp ladder (Life Technologies) on a 2% agarose gel, which was then stained with ethidium bromide and analyzed under UV light. Standard curves were generated for each gene. Relative quantification of gene expression was determined by comparison of threshold values. All samples were analyzed in duplicate in two different dilutions. All results were normalized to GAPDH.

Primer sequences were (5’-3’ forward, reverse):

ATTGTGCCGAGTCCCATATC; AAGCAGGCGTGCATTAGACT (AHR),

CTTGGACCTCTTTGGAGCT; GACCTGCCAATCACTGTG (CYP1A1),

GACGCCTTTATCCTCTCTGCG; ACGACCTGATCCAATTCTGCC (CYP1B1),

CTCTCTGCTCCTCCTGTTCGAC; TGAGCGATGTGGCTCGGCT (GAPDH),

TTCAGTGCTTTGACGTCCTG; TGGAGGAACTGAGCAGCAT (IDO1),

TGCTTCATGCCTTTGATGAG; GAAGGCCTTATGGGAAGGAG (IDO2),

CTCGCCAGTGAAATGATGGCT; GTCGGAGATTCGTAGCTGGAT (IL-1B)

AAATTCGGTACATCCTCGACGG; GGAAGGTTCAGGTTGTTTTCTGC (IL-6),

CGGTGGAAAACACAGATCCA; CTATGCCAGCAGTCTCTTGGG (NAMPT)

GAAGGGTAGTTATCCTGATGCG; CAGAGCTGAGAGAGCGGAA (PAI-2)

TCGATTCGGGACCAGCCCCC; GCTTGCGCACTGCGTTCACC (SOCS3)

AGGAAAAGCAAGCGTAATCTTCA; TATTCCCCGACTGAGCCTGAT (STAT1)

TATGCGGCCAGCAAAGAATCA; CGGGCAATCTCCATTGGCT (STAT3),

GGGAACTACCTGCATTTGGA; GTGCATCCGAGAAACAACCT (TDO).

CACCCTCTAGCAATGTCAACTC; CAGACTCGGGATACTCTCTCC (TIPARP)

Chromatin immunoprecipitation

SKOV-3 cells were exposed to 1 ng/ml IL-6 for 1h or 100µM 4-hydroxytamoxifen for 36h in addition to a corresponding vehicle control. Subsequently, cells were cross-linked for 10 min at 37^0^ C in 1% formaldehyde followed by a quenching step for 10 min with 150 mM glycine. After cross-linking, chromatin DNA was sheared into 200-500 bp fragments by sonication using a Bioruptor®Next Gen (UCD-300, Diagenode SA, Liege, Belgium). Sonicated, soluble chromatin was immune-precipitated with 5 µg of anti-*STAT3* antibody (Santa Cruz Biotechnology, Inc., Heidelberg, Germany), control IPs were performed using rabbit IgG (Millipore, Billerica, MA, USA) corresponding to our specific antibodies. DNA isolates from immunoprecipitates were used as templates for real-time quantitative PCR amplification using the following primer pairs:  *IDO1* forward 5’-GGAGGGGAAACAGGAAACAT-3’, reverse 5’-CTGAGGTTCTGGAATGTGGAG-3’ and *NNMT* forward 5’-TGCAGGTCAGCATTACTTGG-3’, reverse 5’-TTACAGCCTTGTTGCCACTG-3’ as positive control. All ChIP experiments were performed in duplicates.

# siRNA experiments

To knockdown IDO1, STAT1, STAT3, IL-6 and AHR SMART-pool siRNA by Dharmacon RNA Technologies (Lafayette, CO, USA) were used. ON-TARGETplus siCONTROL Non-targeting Pool (D-001810-10-05, Dharmacon) and a transfection without siRNA were used as negative controls.

The target sequences were as follows:

*Human INDO:*

*NM_002164*, sense, 5’-UCACCAAAUCCACGAUCAUUU-3’, antisense, 5’-PUAUGCGAAGAACACUGAAAUU-3’; sense, 5’-UUUCAGUGUUCUUCGCAUAUU-3’, antisense, 5’-PUAUGCGAAGAACACUGAAAUU-3’; sense, 5’-GUAUGAAGGGUUCU GGGAAUU -3’, antisense, 5’-PUUCCCAGAACCCUUCAUACUU-3’; sense, 5’-GAA CGGGACACUUUGCUAAUU-3’, antisense, 5’-PUUAGCAAAGUGUCCCGUUCUU-3’

*Human STAT1, NM_139266*, sense, 5’-GCACGAUGGGCUCAGCUUUUU-3’, antisense, 5’-PAAAGCUGAGCCCAUCGUGCUU-3’; sense, 5’-CUACGAACAUGACCCUAUCUU-3’, antisense, 5’-PGAUAGGGUCAUGUUCGUAGUU-3’; sense, 5’-GAACCUGACUUCCAUG CGGUU-3’, antisense, 5’-PCCGCAUGGAAGUCAGGUUCUU-3’; sense, 5’-AGAAAGAG CUUGACAGUAAUU-3’, antisense, 5’-PUUACUGUCAAGCUCUUUCUUU-3’.

*Human STAT3, NM_213662*, sense, 5’-GAGAUUGACCAGCAGUAUA-3’, antisense, 5’-PUAUACUGCUGGUCAAUCUC -3’; sense, 5’-CAACAUGUCAUUUGCUGAA-3’, antisense, 5’-PUUCAGCAAAUGACAUGUUG-3’; sense, 5’-CCAACAAUCCCAAGAAUGU-3’, antisense, 5’-PACAUUCUUGGGAUUGUUGG-3’; sense, 5’-CAACAGAUUGCCUGCAUUG-3’, antisense, 5’-PCAAUGCAGGCAAUCUGUUG -3’.

*Human IL-6:*

*NM_000600*, sense, 5’-CAAAUUCGGUACAUCCUCG-3’, antisense, 5’-PCGAGGAUGUACCGAAUUUG -3’; sense, 5’-CAGAACAGAUUUGAGAGUA-3’, antisense, 5’- PUACUCUCAAAUCUGUUCUG -3’; sense, 5’-UGAGAAAGGAGACAUGUAA-3’, antisense, 5’-PUUACAUGUCUCCUUUCUCA -3’; sense, 5’-CCACUCACCUCUUCAGAAC-3’, antisense, 5’-PGUUCUGAAGAGGUGAGUGG -3’.

*Human AHR:*

*NM_* *001621*, sense, 5’-GCAAGUUAAUGGCAUGUUU-3’, antisense, 5’-PAAACAUGCCAUUAACUUGC -3’; sense, 5’-GAACUCAAGCUGUAUGGUA-3’, antisense, 5’-PUACCAUACAGCUUGAGUUC-3’; sense, 5’-GCACGAGAGGCUCAGGUUA-3’, antisense, 5’-UAACCUGAGCCUCUCGUGC-3’; sense, 5’-GCAACAAGAUGAGUCUAUU-3’, antisense, 5’-PAAUAGACUCAUCUUGUUGC -3’.

NCI-H596 cells were transfected using lipofectamine RNAiMAX from Invitrogen. Knockdown efficiency was analyzed by qRT-PCR after 24 h or western blot after 48 h. For transfection of SKOV-3 cells, the Amaxa Human Nucleofector Kit V (Amaxa biosystems, Köln, Germany) was used. Briefly, 5 x 10^5^ cells were resuspended in 100 µl of the nucleofector solution and mixed with 1.5 µg of siRNA, then electroporated using program V-005. Cells were immediately transferred into 37°C pre-warmed culture medium (McCOY’s) and plated into 6-well plates, containing 1.5 ml pre-warmed McCOY’s medium. For analysis of kynurenine content in the supernatant, medium was exchanged after 24 h for additional 48 h. The supernatants and the cells were harvested, the knockdown was analyzed by qRT-PCR and the kynurenine and tryptophan content of the medium was measured by HPLC.

shRNA experiments

For knockdown of the AHR in SKOV-3 cells the pSingle-tTS-shRNA vector was purchased from Clontech (CA, USA). Annealed double strand oligonucleotides encoding the desired shRNA sequences with XhoI/HindIII overhangs were cloned into the vector using the XhoI/HindIII cloning sites. shRNA sequences for control/AHR shRNA silencing including the XhoI/HindIII overhangs were as follows:

Non-targeting shRNA antisense oligo 5’-agcttggatccaaaaaagtacttccacctcagttggctctcttgaagccaactg aggtggaagtacc-3’,

Non-targeting shRNA sense oligo 5'-tcgaggtacttccacctcagttggcttcaagagagccaactgaggtggaagta cttttttggatcca-3’,

AHR shRNA antisense oligo (Dharmacon siRNA #6 of Smart pool of AHR siRNA)

5’- agcttggatccaaaaaaggaactcaagctgtatggtatctcttgaataccata cagcttgagttccc-3’,

AHR shRNA sense oligo (Dharmacon)

5’-tcgagggaactcaagctgtatggtattcaagagataccatacagcttgagttc cttttttggatcca-3’.

The recombinant vector was transfected into SKOV-3 cells and clonal transformants were selected with 1 mg/ml neomycin (Sigma-Aldrich). The knockdown was induced using 2 µg/ml doxycyclin (Sigma-Aldrich), cells were analyzed 96 h after induction.

For knockdown of STAT3 in SKOV3 cells shRNA targeting the STAT3 gene with the sense sequence 5’-GCAGCAGCTGA ACAACATGT-3’ was used . A non-targeting shRNA with the sense sequence: 5'-GCCGCTTTGTAGGATAGAG-3' was used for construction of the corresponding shRNA-control transfer vector as previously described . Replication-defective lentiviral particles were produced by transient cotransfection of 7.5 µg pCMVdeltaR8.9, 5 µg pHCMV-G and 10 µg of the pFUGW vector (a kind gift from Dr. D. Baltimore, Bethesda, MD) into a 293T cells with the Lipofectamine 2000 (Invitrogen, Carlsbad, CA) transfection system, as previously described .

# Tissue specimens immunohistochemistry and immunofluorescence

Formalin-fixed paraffin-embedded tissue of human NSCLC, ovarian carcinoma, and frozen tissue of six human NSCLC was provided by the Tissue Bank of the National Center for Tumor Diseases, NSCLC brain metastasis tissue was provided by the Department of Neuropathology, both University Hospital Heidelberg, Germany and used after approval of the local regulatory authorities. Sections cut to 3 µm were processed using a Ventana BenchMark XT® immunostainer (Ventana Medical Systems, Tucson, AZ, USA). The staining procedure included a 1 h pretreatment with either cell conditioner 1 (pH 8) for LCA, IL-6, pSTAT3 and IDO1 or with protease 1 (Ventana) for AHR. The pre-treatment was followed by incubation with either mouse anti-human leukocyte common antigen (LCA) antibody (1:200, Dako, Glostrup, Denmark), rabbit anti-human IL-6 antibody (1:600, Genetex, Irvine, CA, USA), mouse anti-human pSTAT3 antibody (Tyr705, 1:100, Cell Signaling, Danvers, MA, USA), mouse anti-human AHR antibody (1:50, Genetex) or rabbit anti-human IDO1 (1:200, Alexis, Alexis, Lausen, Switzerland) at 37°C for 32 min. Incubation was followed by Ventana standard signal amplification, UltraWash, counterstaining with one drop of hematoxylin for 4 min and one drop of bluing reagent for 4 min. For visualization, ultraView™Universal DAB Detection Kit (Ventana Medical Systems) was used. For quantitative analysis of the IL-6, AHR and pSTAT3 staining patterns, the Histo-Score adapted from Bruna et al. was applied . The score ranges from 0 to 300 and is calculated as the percentage of weakly stained cells plus the percentage of moderately stained cells multiplied by two plus the percentage of strongly stained cells multiplied by three. Quantitative analysis of infiltrating leukocytes was scored by dividing the specimen in four subgroups: negative (0), single, isolated positive cells (1), small groups of positive cells around vessels or single cells apart from vessels (2), and extended areas of positive cell clusters (3). The score for all three proteins was assessed in a 200x magnification field of the area presenting with highest IDO1 expression and of an IDO1 negative area on consecutive sections of each case. Cases homogenously positive or negative were only assessed in one area. For immunofluorescence, slides were deparaffinized according to standard protocols and underwent antigen retrieval for 30 min in cell conditioner 1 (Ventana) in a steam cooker. Primary anti-human pSTAT3 (1:50) anti-human TIPARP (1:50) and anti-human IL-6 (1:50) were incubated overnight at 4°C and detected with AlexaFluor 488nm (1:500, Invitrogen). After application of LinBlock (Linaris, Dossenheim, Germany) blocking solution according to manufacturer’s protocol, slides were incubated with anti-human IDO for 2 h at room temperature and subsequently detected by incubation with AlexaFluor 568nm (1:500, Invitrogen) for 30 min at room temperature.

Western blot analysis

Whole cell lysates were prepared in ice cold tris(hydroxymethyl)aminomethane hydrochloride (TRIS-HCl, 50 mM, pH 8,0; Carl Roth) containing 150 mM NaCl (J.T. Baker, Deventer, Netherlands), 1% NP-40 (AppliChem, Darmstadt, Germany), 10 mM EDTA (Gerbu Biotechnik, Gaiberg, Germany), 200 mM dithiothreitol (DTT, Carl Roth), 100 µM phenylmethylsulphonyl fluoride (PMSF), 10 µg/ml Complete (Roche, Mannheim, Germany) and centrifuged at 4°C (10 min, 13 000 rpm). The protein concentration of the supernatants was determined using the Bio-Rad protein assay (Bio-Rad, Hercules, CA, USA) at 595 nm. The desired amount of protein (20 µg per lane) was separated by 8% SDS-PAGE and transferred to a 0.2 µm-pore nitrocellulose membrane (Whatman, Dassel, Germany). After 1 h of blocking in PBS supplemented with 0.2% Tween 20 (Sigma-Aldrich) and 5% bovine albumin fraction V powder (Carl Roth), the membrane was incubated with rabbit anti-IDO1 antibody (1:2000, Alexis), anti STAT3, anti pSTAT3 (Tyr705), anti STAT1, anti pSTAT1 (Tyr701), anti acetylated lysine (1:1000, Cell Signaling) or goat anti-GAPDH (1:2000, Abcam, Cambridge, UK) as loading control, overnight at 4°C. After a 2 h incubation at room temperature with secondary antibodies donkey anti-rabbit or anti-mouse HRP conjugated (1:5000, GE-Healthcare, Buckinghamshire, UK) or donkey anti-goat HRP conjugated (Santa Cruz Biotechnology), protein detection was performed using ECL Plus reagent (GE Healthcare). Bands were quantified by densitometry using ImageJ software.

Immunoprecipitation

Whole cell lysates were prepared as for western blot analysis and protein content was measured with Bio-Rad protein assay (Bio-Rad, Hercules, CA, USA) at 595 nm. 100 µg of protein lysate was pre-cleared with 10 µl Agarose Protein G beads (Sigma-Aldrich) for 1 h at 4°C on a rotating wheel. 30 µl Agarose Protein G beads (Sigma-Aldrich) were coated with STAT3 antibody (1:100, Cell Signaling, Danvers, MA, USA) or rabbit IgG control (Southern Biotech, Eching, Germany). Pre-cleared lysate was incubated with coated beads overnight at 4°C on a rotating wheel and further analyzed by western blot.

Enzyme Linked Immunosorbent Assay (ELISA)

Human IL-6 was detected with the ELISA Ready-SET-Go Kit (eBioscience, SanDiego, USA) according to manufacturer’s instructions.

# Cocultures of tumor cells and mixed leukocyte reactions (MLR)

2000 SKOV-3 cells or NCI-H596 cells were seeded in flat-bottom 96-well plates in RPMI medium (PAA Laboratories) containing 10% FBS, 100 U/ml penicillin and 100 μg/ml streptomycin. 24 h after seeding 2 * 10^5^ irradiated (30 Gy) PBMC as stimulators and 2 * 10^5^ PBMC from unrelated donors as responders were added in cocultures of tumor cells and MLR. When using AG490, JSI-124 or HATI, cells were pre-incubated with respective agents. After 48 h cells were washed and 2 * 10^5^ irradiated (30 Gy) PBMC as stimulators and 2 * 10^5^ PBMC from unrelated donors as responders were added. When using siIL-6 or scrambled control siRNA, cells were transfected 24 h prior to the addition of PBMC. After 6 days cultures were pulsed with [3H]-methylthymidine (Perkin Elmer, Wellesley, MA, USA) for the last 18 h. The cells were then harvested, and radionuclide uptake was measured by scintillation counting. Pulsed tumor cells only and MLR only served as controls and counts from tumor cells were subtracted from coculture counts. Experiments were repeated with at least 6 unrelated PBMC donors.

Correlation analysis

For correlation analyses of gene expression in mature aggressive B-cell lymphomas (GEO accession number GSE4475) normalised Affymetrix gene expression data were downloaded from the R2 microarray analysis and visualisation platform (http://r2.amc.nl). For IDO1 probeset 210029_at, for AHR probeset 202820_at, for IL-6 probeset 205207_at, for SOCS3 probeset 206360-s-at, for STAT3 probeset 208991_at, for CYP1B1 probeset 202436_s_at, for TIPARP probeset 212665_at, for IL-6R 205945_at, for IL-6ST (gp300) 212195_at and for JAK2 205842_s_at were used.

Spearman rank correlations were computed using Sigmaplot (Systat Software Inc., San Jose, CA, USA). Survival data from NSCLC patients are based on the GEO dataset GSE31210 and were extracted using the PrognoScan database (<http://www.prognoscan.org/>).

Promoter Analysis

The IDO promoter region and potential transcription factors were predicted using the online software Transfac ([https://portal.biobase-international.com](https://portal.biobase-international.com/))

Statistical Analysis

Data are expressed as mean ± s.e.m or as boxplots designed using Sigma Plot. Analysis of significance was performed using the Student’s t-test or Mann-Whitney Rank Sum Test (SigmaPlot). P values < 0.05 were considered significant. Correlations were analysed by Spearman rank correlation (Sigmaplot).

**Supplemental References**

Anastasov N, Bonzheim I, Rudelius M, Klier M, Dau T, Angermeier D, Duyster J, Pittaluga S, Fend F, Raffeld M, Quintanilla-Martinez L: C/ebpbeta expression in alk-positive anaplastic large cell lymphomas is required for cell proliferation and is induced by the stat3 signaling pathway. Haematologica;95:760-767.

Anastasov N, Klier M, Koch I, Angermeier D, Hofler H, Fend F, Quintanilla-Martinez L: Efficient shrna delivery into b and t lymphoma cells using lentiviral vector-mediated transfer. J Hematop 2009;2:9-19.

Bowers EM, Yan G, Mukherjee C, Orry A, Wang L, Holbert MA, Crump NT, Hazzalin CA, Liszczak G, Yuan H, Larocca C, Saldanha SA, Abagyan R, Sun Y, Meyers DJ, Marmorstein R, Mahadevan LC, Alani RM, Cole PA: Virtual ligand screening of the p300/cbp histone acetyltransferase: Identification of a selective small molecule inhibitor. Chem Biol 2010;17:471-482.

Bruna A, Darken RS, Rojo F, Ocana A, Penuelas S, Arias A, Paris R, Tortosa A, Mora J, Baselga J, Seoane J: High tgfbeta-smad activity confers poor prognosis in glioma patients and promotes cell proliferation depending on the methylation of the pdgf-b gene. Cancer Cell 2007;11:147-160.

Fritsche E, Schafer C, Calles C, Bernsmann T, Bernshausen T, Wurm M, Hubenthal U, Cline JE, Hajimiragha H, Schroeder P, Klotz LO, Rannug A, Furst P, Hanenberg H, Abel J, Krutmann J: Lightening up the uv response by identification of the arylhydrocarbon receptor as a cytoplasmatic target for ultraviolet b radiation. Proc Natl Acad Sci U S A 2007;104:8851-8856.

Gao LF, Xu DQ, Wen LJ, Zhang XY, Shao YT, Zhao XJ: Inhibition of stat3 expression by sirna suppresses growth and induces apoptosis in laryngeal cancer cells. Acta Pharmacol Sin 2005;26:377-383.

Hummel M, Bentink S, Berger H, Klapper W, Wessendorf S, Barth TF, Bernd HW, Cogliatti SB, Dierlamm J, Feller AC, Hansmann ML, Haralambieva E, Harder L, Hasenclever D, Kuhn M, Lenze D, Lichter P, Martin-Subero JI, Moller P, Muller-Hermelink HK, Ott G, Parwaresch RM, Pott C, Rosenwald A, Rosolowski M, Schwaenen C, Sturzenhofecker B, Szczepanowski M, Trautmann H, Wacker HH, Spang R, Loeffler M, Trumper L, Stein H, Siebert R: A biologic definition of burkitt's lymphoma from transcriptional and genomic profiling. N Engl J Med 2006;354:2419-2430.

Lee JE, Safe S: 3',4'-dimethoxyflavone as an aryl hydrocarbon receptor antagonist in human breast cancer cells. Toxicol Sci 2000;58:235-242.

Okayama H, Kohno T, Ishii Y, Shimada Y, Shiraishi K, Iwakawa R, Furuta K, Tsuta K, Shibata T, Yamamoto S, Watanabe S, Sakamoto H, Kumamoto K, Takenoshita S, Gotoh N, Mizuno H, Sarai A, Kawano S, Yamaguchi R, Miyano S, Yokota J: Identification of genes upregulated in alk-positive and egfr/kras/alk-negative lung adenocarcinomas. Cancer Res 2012;72:100-111.

Opitz CA, Litzenburger UM, Lutz C, Lanz TV, Tritschler I, Koppel A, Tolosa E, Hoberg M, Anderl J, Aicher WK, Weller M, Wick W, Platten M: Toll-like receptor engagement enhances the immunosuppressive properties of human bone marrow-derived mesenchymal stem cells by inducing indoleamine-2,3-dioxygenase-1 via interferon-beta and protein kinase r. Stem Cells 2009;27:909-919.

Schmitt M, Pawlita M: High-throughput detection and multiplex identification of cell contaminations. Nucleic Acids Res 2009;37:e119.

Weksler BB, Subileau EA, Perriere N, Charneau P, Holloway K, Leveque M, Tricoire-Leignel H, Nicotra A, Bourdoulous S, Turowski P, Male DK, Roux F, Greenwood J, Romero IA, Couraud PO: Blood-brain barrier-specific properties of a human adult brain endothelial cell line. FASEB J 2005;19:1872-1874.

Yue EW, Douty B, Wayland B, Bower M, Liu X, Leffet L, Wang Q, Bowman KJ, Hansbury MJ, Liu C, Wei M, Li Y, Wynn R, Burn TC, Koblish HK, Fridman JS, Metcalf B, Scherle PA, Combs AP: Discovery of potent competitive inhibitors of indoleamine 2,3-dioxygenase with in vivo pharmacodynamic activity and efficacy in a mouse melanoma model. J Med Chem 2009;52:7364-7367.
